# Endorobotic submucosal dissection using the da Vinci SP system: a 101-case experience in robotic transanal surgery

**DOI:** 10.1007/s00464-026-12671-2

**Published:** 2026-03-05

**Authors:** Metincan Erkaya, Salih Karahan, Mustafa Oruc, Ali Alipouriani, Kamil Erozkan, Ilker Ozgur, Scott R. Steele, Joshua Sommovilla, Emre Gorgun

**Affiliations:** Department of Colorectal Surgery, Digestive Disease & Surgery Institute, Cleveland Clinic, 9500 Euclid Avenue, Cleveland, OH 44195 USA

**Keywords:** Robotic surgery, Single-port robot (SP), Endoscopic submucosal dissection, Non-malignant polyps, Pathological outcomes

## Abstract

**Background:**

Endorobotic submucosal dissection (ERSD) using the da Vinci single-port (SP) platform offers high-definition visualization and enhanced precision for the resection of distal colorectal lesions. In contrast, conventional endoscopic submucosal dissection is technically demanding and associated with a steep learning curve. This study aims to evaluate the efficacy, broader applicability, and long-term outcomes of ERSD in a large patient cohort.

**Methods:**

We retrospectively analyzed 101 patients who underwent ERSD using the da Vinci SP platform between March 2020 and May 2025. Patient demographics, lesion characteristics, procedural details, pathological findings, and long-term follow-up data were reviewed. The primary objectives were to assess intraoperative and postoperative complications, evaluate the feasibility of en-bloc submucosal dissection, and examine the oncological outcomes associated with the ERSD procedure.

**Results:**

The median age of the cohort was 62.5 years (IQR: 52–70 years), with 56.4% male patients. The median distance from the anal verge was 8 cm (IQR: 6–11 cm, range: 1–24 cm). En-bloc resection was achieved in 96.0% of the cases, with a median procedure time of 73 min (IQR: 53–97). The median length of hospital stay was 0 days. The median specimen size was 19 cm^2^ (IQR: 11–28), with a median greatest dimension of 5 cm (range: 1–18 cm). Final pathology revealed tubulovillous adenoma in 57.4% of the cases, tubular adenoma in 16.7%, and serrated adenoma in 6.0%, adenocarcinoma in 12.9%, neuroendocrine tumor in 1.0%, villous adenoma in 1.0%, and colonic mucosa/scar in 5.0%. No metastases or malignant recurrences were observed during the median follow-up of 22 months in patients with adenocarcinoma. Across the entire cohort, three non-cancer-related deaths occurred during follow-up.

**Conclusion:**

Our experience demonstrates that ERSD is not only safe and feasible but also provides durable results with minimal long-term complications and high rates of en-bloc resection in distal colorectal lesions. These findings support the broader application of ERSD as a viable alternative to the traditional transanal approaches for selected colorectal lesions.

**Supplementary Information:**

The online version contains supplementary material available at 10.1007/s00464-026-12671-2.

Transanal surgery has evolved as a crucial organ-preserving approach for the treatment of patients with benign and early malignant rectal lesions, thereby avoiding the significant morbidity and functional impairment of radical surgical interventions [1, 2]. However, despite these advantages, conventional transanal techniques encounter significant technological limitations. A principal challenge in endoluminal surgery is platform instability, which compromises precision during dissection. Furthermore, standard colonoscopes equipped with a single working channel hinder critical procedures such as traction and triangulation. These technical constraints have led to the development of advanced surgical platforms.

Robotic transanal surgery has emerged as a promising innovation in this field [3, 4]. The integration of robotic systems, particularly the da Vinci platforms, has enhanced surgical precision. While the da Vinci Xi system advanced robotic transanal capabilities, concerns persisted regarding potential external sphincter damage from multiport systems and challenges with instrument maneuverability in confined operative fields. These limitations necessitated the development of more specialized platforms [5]. The subsequent introduction of the da Vinci single-port (SP) robotic platform effectively addressed many of these limitations, enabling wider clinical adoption of robotic platforms for transanal procedures [6, 7].

EndoRobotic Submucosal Dissection (ERSD) represents a notable advancement, integrating the principles of submucosal dissection with robotic transanal techniques utilizing the da Vinci SP system. Although previous studies have demonstrated the safety and feasibility of Robotic Transanal Minimally Invasive Surgery (R-TAMIS), [8–11] ERSD presents distinct advantages over R-TAMIS. In contrast to R-TAMIS, which typically involves full-thickness resection and may consequently increase the risk of luminal narrowing, ERSD allows precise dissection within the submucosal plane. This approach potentially preserves the rectal reservoir function, thereby minimizing postoperative bowel symptoms [12, 13].

Despite the potential advantages of ERSD, comprehensive clinical data regarding this technique remain limited. The primary objective of this study was to assess the long-term complications and oncological outcomes associated with ERSD by analyzing the largest patient cohort to date. Through this evaluation, we aimed to provide substantive insights into the clinical utility of this method for distal colorectal lesions and its potential role in the evolving landscape of robotic transanal surgery while establishing a foundation for the clinical adoption of this technique.

## Methods

### Patient selection

We conducted a retrospective analysis of an IRB-approved prospectively collected database from a single institution. All patients who underwent elective ERSD using the da Vinci SP system between March 2020 and May 2025 were included in the study. As a tertiary referral center for endoluminal procedures, all patients had undergone prior colonoscopy with tissue biopsy. The inclusion criteria were aligned with standard endoscopic submucosal dissection guidelines. Patients eligible for ERSD procedures had target lesions located within 24 cm of the anal verge.

The procedure was indicated for benign neoplasms, pre-cancerous lesions with high-grade dysplasia, and suspicious lesions that were endoscopically concerning but had no evidence of invasive adenocarcinoma on pre-operative biopsy. Patient selection and technical approach were individualized based on multidisciplinary tumor board evaluation (MDTB). For lesions with benign histology on pre-operative biopsy and no suspicious morphological features on endoscopic assessment, standard submucosal dissection was performed. For lesions that demonstrated concerning features on endoscopic and radiologic evaluation, outside biopsy specimens were re-reviewed when indicated, and the depth of excision was tailored accordingly. Suspected lesions underwent either intermuscular dissection or full-thickness excision depending on the suspected depth of invasion.

Based on the final pathology results, patients either underwent salvage surgery or were placed on surveillance protocols as determined by the multidisciplinary tumor board (MDTB) in collaboration with the patient. Patients with poor prognostic features including deep submucosal invasion, positive margins, perineural invasion, lymphovascular invasion, poor differentiation, or high-grade tumor budding were counseled regarding salvage surgery.

### The da Vinci SP platform

The da Vinci SP robotic system is a minimally invasive surgical platform that is designed for endoluminal procedures. This platform features a single 25 mm cannula housing three double-jointed, fully articulating instruments and a flexible 3D camera. The system’s unique "C-shaped" arm design allows for 360-degree rotation around the cannula’s center and within the instrument port, facilitating comprehensive access to all quadrants of the rectum without repositioning the patient or redocking the robot. The SP’s camera moves in a serpiginous fashion over the target lesions, providing additional degrees of freedom to clearly visualize the proximal margins. The SP console incorporates a holographic display that provides surgeons and assistants with real-time instrument positioning and orientation information. A distinctive foot pedal enables the coordinated movement of the camera and instruments as a single unit, enhancing surgical precision in confined spaces. The platform’s articulated instruments offer improved triangulation around the target anatomy, facilitating more effective dissection, suturing, and tissue manipulation than traditional transanal platforms. While the SP system presents some limitations, such as incompatibility with certain instruments and a steep learning curve, its potential to improve visualization, reduce instrument collisions, and protect anal sphincters makes it a promising tool for advancing transanal surgical techniques. Future enhancements may further expand its applicability to more proximal colorectal lesions, thereby broadening the scope of robot-assisted endoluminal surgeries. (Supplemental Fig. 1).

**Fig. 1:**
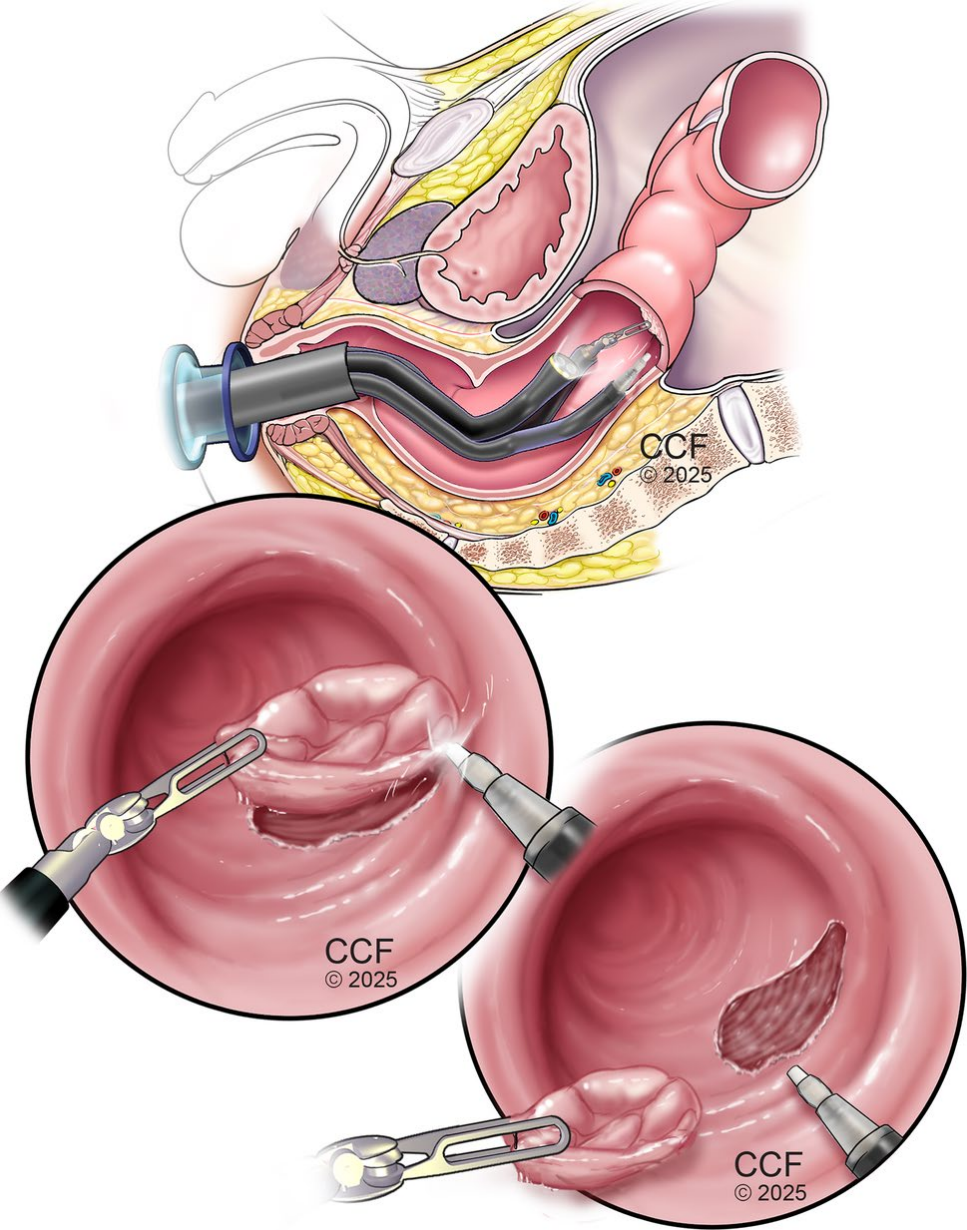
EndoRobotic submucosal dissection

### Basic terminology

We use the term EndoRobotic Submucosal Dissection (ERSD) to specifically describe a technique that combines the principles of endoscopic submucosal dissection with a robotic transanal approach using the da Vinci SP platform. Unlike similar approaches such as R-TAMIS, which typically involves full-thickness resection, ERSD represents a distinct methodology that utilizes hypertonic saline solution to create a cushion within the submucosal layer for submucosal plane dissection while preserving the integrity of underlying rectal wall integrity.

### Operative intervention

**Fig. 2:**
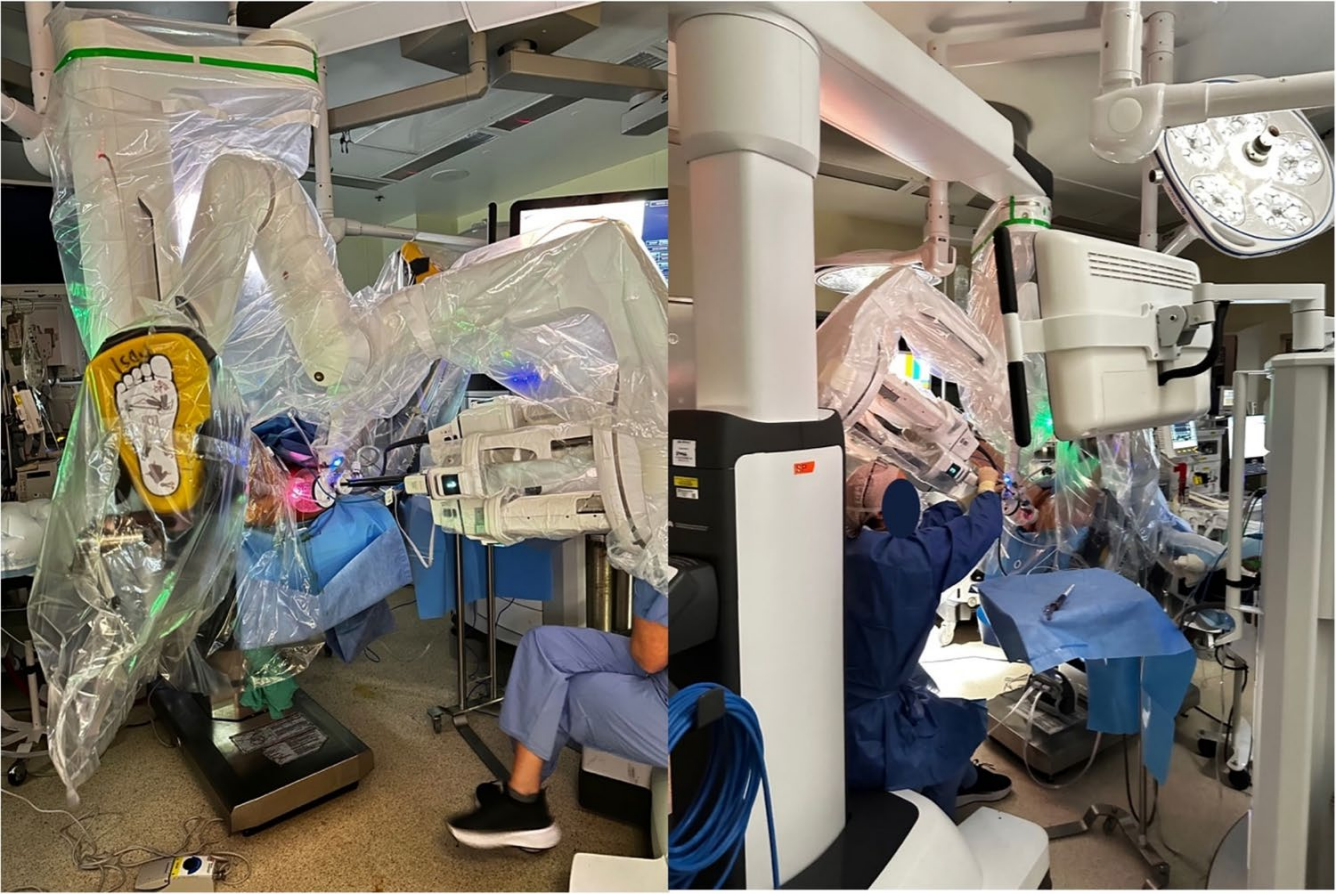
Operating room setup: da Vinci SP docking and patient positioning

All procedures were performed using our previously described ERSD technique via the da Vinci SP platform with minor refinements to the transanal access port [12, 13] (Fig. 1). The patients were positioned in a modified lithotomy with a slight Trendelenburg tilt. Following the digital rectal examination, the da Vinci SP robot was docked from the patient’s left side, maintaining direct access to the perineum for the surgeon or assistant (Fig. 2).

**Fig. 3:**
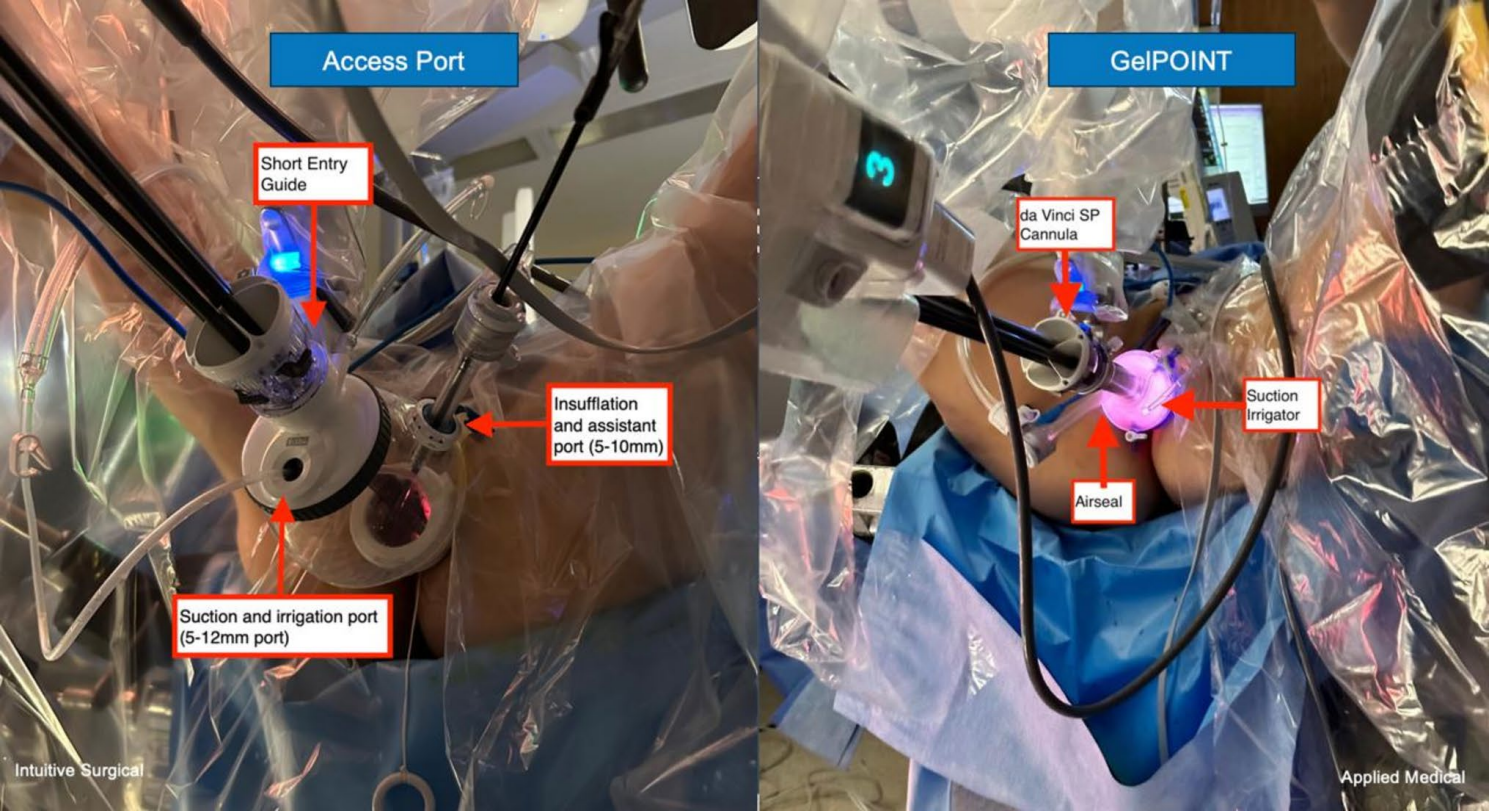
Transanal access platforms: **a** GelPOINT Path (Applied Medical, Rancho Santa Margarita, CA, USA); **b** da Vinci SP access port (Intuitive Surgical Inc., Sunnyvale, CA, USA)

Initially, GelPOINT Path (Applied Medical, Rancho Santa Margarita, CA, USA) was used as the transanal access port. Subsequently, when the da Vinci SP access port (Intuitive Surgical Inc., Sunnyvale, CA, USA) became available specifically for transanal procedures, the new system was fully implemented. The da Vinci SP access port consists of a wound retractor and a transparent chamber (‘bubble’) with a multichannel cannula for robotic instruments. This access port includes a laparoscopic port (5–10 mm) for insufflation and assistant access along with a remotely operated port (5–12 mm) for suction and irrigation (Fig. 3). The da Vinci SP port was inserted after transanal access port placement.

**Fig. 4:**
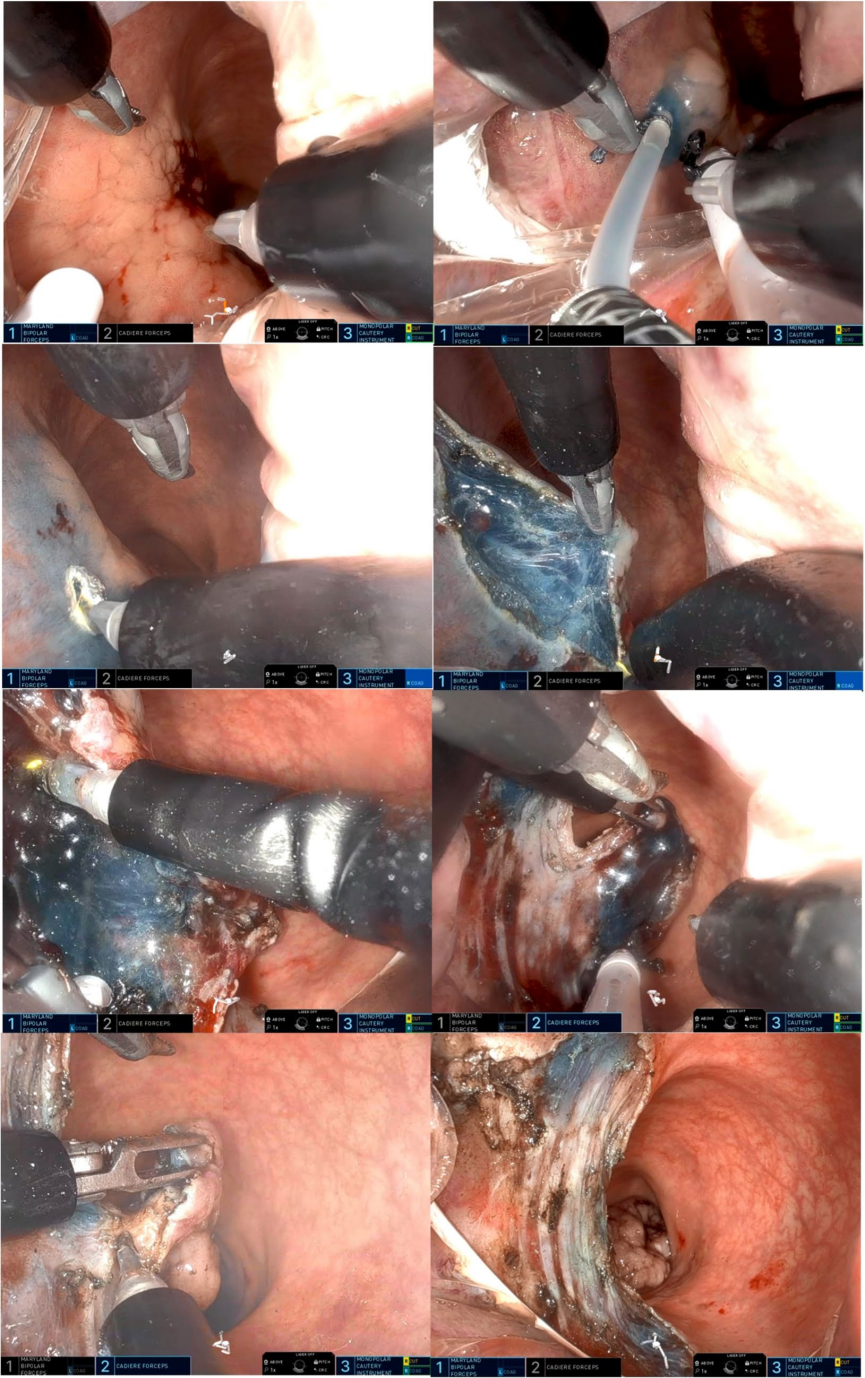
Sequential steps in endorobotic submucosal dissection (ERSD)

**Fig. 5:**
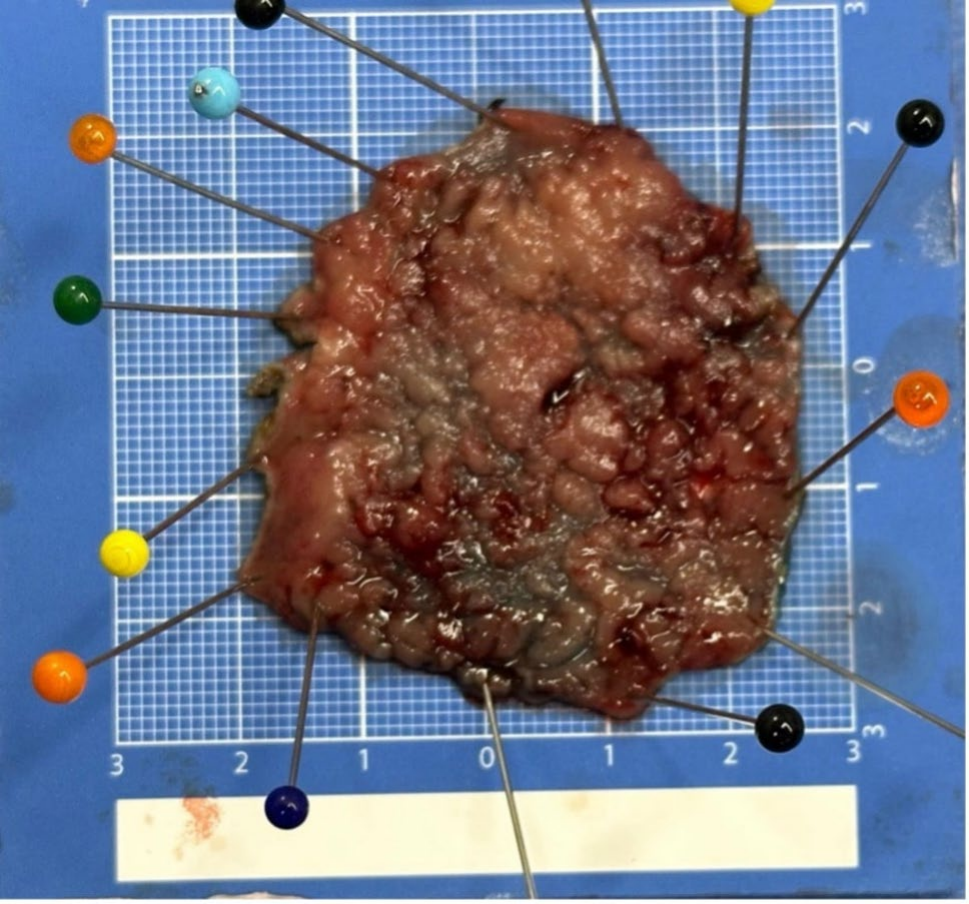
En Bloc resected specimen pinned on corkboard

Dissection was performed in the submucosal plane following the establishment of an adequate pneumorectum and submucosal lifting using a robotic monopolar spatula-tip device. Escalation to full-thickness excision was performed when intraoperative findings indicated suspected deep mural invasion or inability to maintain a safe submucosal plane. Upon completion of the resection, the surgical site was thoroughly inspected for perforation or bleeding. The decision to close the defect was individualized based on intraoperative findings. In cases with intact muscularis propria and no active bleeding, the defect was preferentially left open to heal by secondary intention, particularly for large circumferential lesions where primary closure might increase stricture risk (Fig. 4). However, when active bleeding was observed or any breach in the muscularis was detected, closure was prioritized using barbed knotless sutures (V-Loc, Medtronic, MN) in a transverse sewing pattern or endoclips was placed to prevent delayed bleeding or perforation. Following completion, the robot was undocked, and the specimen was extracted for en-bloc pathological examination (Fig. 5).

## Results

### Patient demographics and lesion characteristics

A total of 101 patients underwent ERSD using the da Vinci Single-Port platform between March 2020 and May 2025. The median age was 62.5 years (IQR: 52–70 years), with 57 males (56.4%) and 44 females (43.6%). Median BMI was 27.6 kg/m^2^ (IQR: 24.7–31.6). Sixty-three patients (62.4%) were classified as ASA III-IV, and thirty-eight (37.6%) were ASA I-II. Most lesions were lateral (30.7%), followed by posterior (20.7%), posterolateral (14.9%), anterior (14.9%), anterolateral (11.9%), and circumferential (6.9%) lesions. The median distance from the anal verge was 8 cm (IQR: 6–11 cm), with a range of 1–24 cm (Table 1).

**Table 1:** Patient demographics and perioperative characteristics

Category	ERSD (*n* = 101)
Demographic	
Age, years	62.5 (52 – 70)
Gender, male	57 (56.4%)
BMI, kg/m^2^	27.6 (24.7 – 31.6)
ASA score	
I – II	38 (37.6%)
III – IV	63 (62.4%)
Pre-operative characteristics	
Lesion location	
Anterolateral	12 (11.9%)
Anterior	15 (14.9%)
Circumferential	7 (6.9%)
Lateral	31 (30.7%)
Posterior	21 (20.7%)
Posterolateral	15 (14.9%)
Distance to anal verge, cm (IQR)	
Median (IQR)	8 (6 – 11)
Range	[1–23]
Intra-operative characteristics	
Operation time, min	73 (53 – 97)
Conversion rate	
Transabdominal	0 (0)
Transanal	6 (5.9%)
Defect closure	
No closure	49 (48.5%)
Suture closure	32 (31.7%)
Suture closure with clip	20 (19.8%)
Estimated blood loss, ml	5 (1 – 5)
Perforation	2 (2.0%)
Post-operative characteristics	
En-bloc resection	97 (96.0%)
Specimen size, cm^2^	19 (11—28)
Greatest dimension, cm [range]	5 [1–18]
Length of hospital stay, day	0 (0–0)
Complications	
Urinary retention/UTI	2 (2.0%)
Bleeding	6 (5.9%)
Perforation	1 (1.0%)
Pathology	
Tubulovillous adenoma	58 (57.4%)
Tubular adenoma	17 (16.7%)
Serrated adenoma	6 (6.0%)
Neuroendocrine tumor	1 (1.0%)
Villous adenoma	1 (1.0%)
Adenocarcinoma	13 (12.9%)
Colonic mucosa/Scar	5 (5.0%)

Data are presented as median (IQR) for continuous variables and as percentages for categorical variables

*BMI* Body mass index, *ASA* American Society of Anesthesiologists, *UTI* Urinary tract infection, *IQR* Interquartile range

### Procedural outcomes

The median procedure time was 73 min (IQR: 53–97). En-bloc resection was achieved in 97 patients (96.0%). Conversion was required in six cases (5.9%), where the procedure was converted to full-thickness R-TAMIS due to bulky tumor characteristics that made standard ERSD technically challenging. The median estimated blood loss was 5 mL (IQR: 1–5). For defect management, 49 patients (48.5%) did not require closure, 32 patients (31.7%) underwent suture closure, and 20 patients (19.8%) received suture closure with clips. The median specimen size was 19 cm^2^ (IQR: 11–28), with a median greatest dimension of 5 cm (range: 1–18 cm). The majority of patients were discharged on the same day as their surgery. Two patients had intraoperative full-thickness defects. The first case involved a 15-cm circumferential carpeting polyp extending from the dentate line with a defect on the left lateral wall and the other on the posterior wall. Both defects were repaired with V-Loc sutures and the patients were managed postoperatively without complications. One patient developed with bleeding on postoperative day 4 and computed tomography showed evidence of intra-abdominal air, suggestive of a full-thickness defect. Flexible sigmoidoscopy confirmed this finding and the defect was successfully managed using endoscopic clipping. The patient was subsequently discharged in stable condition. Furthermore, overall postoperative complications were minimal, with urinary retention/urinary tract infection occurring in two (2.0%) patients and late bleeding observed in six (5.9%) patients. Most complications were managed conservatively; however, one patient required transfusion of three units of blood despite no active bleeding source being identified during endoscopy.

### Pathological findings

The final pathological diagnosis included: tubulovillous adenoma in 58 patients (57.4%), tubular adenoma in 17 patients (16.8%), serrated adenoma in 6 patients (6.0%), adenocarcinoma in 13 patients (12.9%), neuroendocrine tumor in 1 patient (1.0%), villous adenoma in 1 patient (1.0%), and colonic mucosa/scar in 5 patients (5.0%). Among the 14 patients with malignant lesions (13 adenocarcinomas and 1 neuroendocrine tumor), 10 patients (71.4%) achieved negative margins for malignancy. Four patients (28.6%) had positive margins: three with deep margin involvement and one requiring conversion to full-thickness R-TAMIS because due of to the inability to achieve en-bloc resection.

### Management and follow-up of malignant cases

MDTB recommendations resulted in the following treatments: low anterior resection in six patients, total proctocolectomy in one patient, additional local excision in one patient, and active surveillance in five patients. One patient had a stroke and was lost to follow-up before definitive surgical management could be implemented. Final pathology from patients who underwent subsequent surgery revealed no residual tumor (pT0N0) in any specimen except one, which demonstrated pT3N1b disease. During a median follow-up of 22 months for patients with adenocarcinoma, no local recurrence or distant metastases were observed. Across the entire cohort, three non-cancer-related deaths occurred during follow-up, including two due to septicemia and one due to cardiogenic shock. No procedure-related mortalities occurred. Detailed preoperative variables of the patients with pathologically confirmed malignant cases are presented in Table 2, including patient demographics, lesion characteristics, and preoperative diagnoses. The histopathological features of these malignant cases, including margin status, TNM staging, and histological findings, are outlined in Table 3.

**Table 2:** Preoperative variables of patients with pathologically confirmed cancer

Pt #	Age	Sex	BMI	ASA score	Specimen surface (cm^2^)	Location	Distance to AV (cm)	Procedure time (min)	Preoperative diagnosis
1	70	M	29,7	2	19	Posterolateral	10	97	TVA
2	70	M	29.6	3	20.3	Lateral	9	113	TVA w/HGD
3	80	F	24,3	3	4.9	Posterolateral	8	90	IMCa w/ HGD
4	51	F	42,6	3	18.6	Anterior	13	88	TVA w/HGD
5	68	M	24,4	2	3.5	Anterior	6	53	TVA w/HGD
6	46	M	34.3	3	3.8	Anterior	7	38	NET
7	78	M	29.1	2	150	Posterolateral	10	226	TVA w/HGD
8	33	F	18,8	2	5.9	Posterior	10	58	Chronic colitis w/HGD
9	66	F	17,9	3	11.8	Anterior	2	30	TVA
10	53	F	29,8	2	25.2	Anterior	15	70	TVA
11	68	M	24,9	2	27.5	Lateral	5	60	TVA
12	59	F	33,1	2	52.3	Lateral	10	107	TVA
13	70	F	22.3	3	9.2	Posterolateral	5	63	TVA
14	62	M	42,3	3	8,9	Anterior	18	86	TVA

*HGD* High-Grade Dysplasia, *ASA* American Society of Anesthesiologists Score, *BMI* Body Mass Index, *TVA* Tubulovillous adenoma, *IMCa* Intramucosal Carcinoma, *NET* Neuroendocrine tumor

**Table 3:** ERSD pathology reports of patients with pathologically confirmed cancer

	ERSD Pathology Report										MDTB decision / action	Final pathology	Follow-up / surveillance results
Pt #	Any margin involvement for carcinoma	Pathology	pT stage	Deep resection margin (mm)	SMI depth (mm)	PNI	LVI	Grade	Size (cm)	Budding			
1	Negative	Adenoca	pT1	0.41	SM2	No	No	G2	0.8	BD1	Re-excision	pT0	No R/M
2	Negative	Adenoca	pT1	0.93	SM3	N/A	No	G2	N/A	BD2	Surveillance		No R/M
3	Present (deep margin)^✦^	Adenoca	pT1	0	SM3	No	No	G2	1.2	BD1	LAR	pT0N0	No R/M
4	Negative	Adenoca	pT1	0.27	SM3	No	Yes	G2	0.25	BD2	LAR	pT0N0	No R/M
5	Negative	Adenoca	pT1	1.5	SM3	Yes	No	G2	0.6	BD1	LAR	pT0N0	No R/M LAR syndrome
6	Negative^†^	NET	pT1a	0.8	N/A	N/A	N/A	G1	0.6	N/A	Surveillance		No R/M
7	N/A^✦b^	Adenoca	pTis	2	N/A	N/A	N/A	N/A	0.7	N/A	Surveillance		No R/M Rectal stricture
8	Negative^a^	IMCa	pTis	2	N/A	No	No	N/A	0.55	N/A	Total proctocolectomy with IPAA	pT0N0	No R/M
9	Negative^a^	Adenoca	pT1	< 1	SM1	Yes	No	G2	0.125	BD3	*		
10	Negative	Adenoca	pT1	1.75	SM1	No	No	G2	0.1	BD1	Surveillance		No R/M
11	Negative	Adenoca	pT1	7	SM1	No	No	G2	0.1	BD1	Surveillance		No R/M
12	Present (deep margin)^✦^	Adenoca	pT1	< 1	SM1	No	No	G1	N/A	BD1	LAR	pT0N0	No R/M
13	Negative^✦a^	Adenoca	pT2	2	N/A	No	No	G2	1.4	BD2	LAR	pT0N0	No R/M
14	Present (deep margin)^✦^	Adenoca	pT2	0	N/A	No	No	G2	3.2	BD3	LAR	pT3N1b	No R/M

^a^Margins are negative for carcinoma, but low-grade dysplasia is present at the mucosal resection margin

^b^En bloc resection not achieved

^*^Surgery was not performed as the patient experienced a stroke and was lost to follow-up, preventing further surgical planning

^✦^Transanal full-thickness excision was performed

^†^Intermuscular dissection performed for scar site excision following prior polypectomy with positive margins

*APR* Abdominoperineal resection, *LAR* Low anterior resection, *NET* Neuroendocrine tumor, *No R/M* No recurrence or metastasis, *PNI* Perineural invasion, *LVI* Lymphovascular invasion, *SMI* Submucosal invasion, *MDTB* Multidisciplinary tumor board, *IMCa* Intramucosal carcinoma,

## Discussion

In this series spanning over five years, we present our institutional experience with ERSD using the da Vinci Single-Port platform, demonstrating favorable clinical and oncologic outcomes for distal colorectal lesions with high technical success rates. Our en-bloc resection rate was 96.0%, which exceeds the rates reported for endoscopic submucosal dissection (ESD), typically ranging from 70 to 90% [14–17]. This high en-bloc resection rate is critical for accurate histopathological assessment of malignant lesions, enabling precise determination of invasion depth, margin status, and adverse histopathological features. Furthermore, the absence of local recurrence and distant metastasis during follow-up, along with a high rate of negative margins in malignant lesions, support the favorable oncological profile of this approach in selected cases.

The technical advantages of ERSD over the conventional endoscopic approaches are notable. While traditional ESD can be effective in the hands of experienced surgeons, it is limited by restricted instrument triangulation, suboptimal ergonomics, and insufficient platform stability. Moreover, traditional ESD requires a steep learning curve, typically more than 100 procedures, with full mastery potentially requiring approximately 250 cases [18–20]. Surgeons with prior robotic experience may transition to the ERSD technique more readily, potentially expanding the application of submucosal dissection. This adaptability could facilitate broader adoption of advanced endoluminal techniques, particularly in Western settings where conventional ESD remains less common [21].

The SP robotic platform addresses many limitations of conventional endoscopy by providing high-definition 3D visualization, wristed instrument articulation for precise dissection within the narrow rectal lumen, and improved ergonomics for the operator. These advantages may have contributed to the high technical success rate even in the presence of challenging lesions. Previous studies have shown that the SP robotic platform is feasible for a broad spectrum of colorectal procedures, with favorable clinical and oncological outcomes [9, 22]. Since its introduction, transanal robotic surgery for rectal lesions has continued to evolve, with R-TAMIS gaining widespread adoption because of its lower morbidity compared to more radical excision techniques [8–10]. Early clinical experience with the SP platform has demonstrated favorable outcomes, with a phase II trial of 26 patients achieving 100% en-bloc resection rates and only 15.4% complications [23]. A recent systematic review of 18 studies including 317 patients reported an overall complication rate of 9.7% for R-TAMIS [24]. Comparative analyses have demonstrated that R-TAMIS achieves faster operative times with comparable clinical and oncologic outcomes and significantly lower postoperative morbidity rates than transanal endoscopic microsurgery [25]. R-TAMIS has demonstrated the capability to achieve en‑bloc resection of low-risk rectal tumors while maintaining high rates of negative margins and low complication rates [23, 26–28]. From an oncologic perspective, R-TAMIS for early rectal cancer has demonstrated favorable short-term outcomes with 90% R0 resection rates, predominantly pT1 disease (55%), and only 5% local recurrence in appropriately selected patients [29]. Our findings are consistent with this growing body of evidence. Among the 14 patients with final malignant pathology in our series, negative resection margins were achieved in 10 cases (71%). These results highlight the potential value of ERSD as both a diagnostic tool providing complete pathologic assessment and an organ-preserving therapeutic option for early distal colorectal malignancies when strict postoperative surveillance and multidisciplinary management are maintained.

In addition to favorable oncologic outcomes, ERSD demonstrated significant efficiency advantages, with a median operative time of only 73 min (IQR: 53–97 min). This efficiency is clinically meaningful given that our study cohort included predominantly challenging lesions that may be unsuitable for conventional local excision techniques. The median lesion size was 5 cm (range 1–18 cm), which exceeded the standard definition of large colorectal lesions (≥ 2 cm) [30, 31]. Additionally, many lesions presented with extensive circumferential coverage of the lumen, adding complexity to the resection process. Despite these challenges, we achieved a high en-bloc resection rate, highligting the potential clinical advantages of this approach for complex distal colorectal lesions.

Functional outcomes following transanal minimally invasive approaches remain underexplored [32]. Nevertheless, Goldenshluger et al. [33] reported that 70% of patients experienced good postoperative flatus control and 82% demonstrated favorable stool control following TAMIS. Additionally, low anterior resection syndrome was classified as mild in 17% of patients and major in only 9%. An important consideration when selecting between submucosal dissection and full-thickness excision for lesions with uncertain malignant potential is the impact on salvage surgery if invasive cancer is identified on final pathology. Full-thickness local excision techniques such as TAMIS breach the muscularis propria, which may cause tissue reactions and potentially disrupt the total mesorectal excision (TME) planes in cases requiring subsequent radical resection. Such disruption may necessitate a greater stapling distance below the lesion, increasing the risk of permanent colostomy, higher complication rates, and prolonged operative times due to suboptimal surgical planes. These concerns are supported by the GRECCAR 2 trial [34] which reported significantly higher complication rates (46% versus 22%) and permanent colostomy rates (25% versus 9%) following completion TME after local excision. Furthermore, two meta-analyses demonstrated increased operative times in patients who underwent TME after local excision. Conversely, salvage surgery after colorectal ESD has shown postoperative morbidity rates comparable to those of primary surgery [35, 36]. Therefore, ERSD represents a potential alternative for lesions with suspected malignancy or T1 features on preoperative biopsy, which are candidates for endoscopic removal. This approach could potentially reduce complications and improve clinical outcomes by enabling precise submucosal dissection while preserving the deeper tissue layers.

Patient selection remains critical for optimal outcomes of ERSD. Our experience suggests that ERSD may offer advantages for lesions in anatomically challenging locations where conventional endoscopic approaches are limited by restricted instrument triangulation and suboptimal visualization. The ability to resect larger lesions while maintaining high en-bloc resection rates may distinguish ERSD from traditional techniques. This could be valuable when malignancy is suspected as ERSD facilitates complete specimen removal while potentially maintaining oncological safety. Moreover, our study demonstrated minimal complications, suggesting that ERSD may be a suitable alternative for select high-risk patients, including those with prior procedures, scarring, or post-chemoradiation treatment.

Despite these promising results, several limitations must be acknowledged. First, this was a retrospective, single-center study without a control group. Second, while our median follow-up of 22 months for malignant cases provides initial oncological data, a longer follow-up period is needed to confirm the durability of these oncological outcomes. Third, the procedure requires specialized expertise; surgeons performing ERSD should possess advanced proficiency in both robotic systems and transanal surgical techniques. Additionally, the development of purpose-built instruments specifically designed for endoluminal robotic surgery could further enhance the capabilities of this technique.

## Conclusion

Our experience over a five-year period with ERSD using the da Vinci SP platform demonstrates that this technique is a safe and effective alternative for the resection of distal colorectal lesions, achieving high en-bloc resection rates with minimal complications. The favorable clinical and oncological outcomes support its potential for broader adoption in advanced endoluminal surgery.

## Supplementary Information

Below is the link to the electronic supplementary material.

Supplementary file1 (JPG 135 KB)
