# Supplementary figures and images for: Endorobotic submucosal dissection using the da Vinci SP system: a 101-case experience in robotic transanal surgery
Source: Surg Endosc. 2026 Mar 5;40(5):4136–46. doi: 10.1007/s00464-026-12671-2 (PMC13161288; doi:10.1007/s00464-026-12671-2)

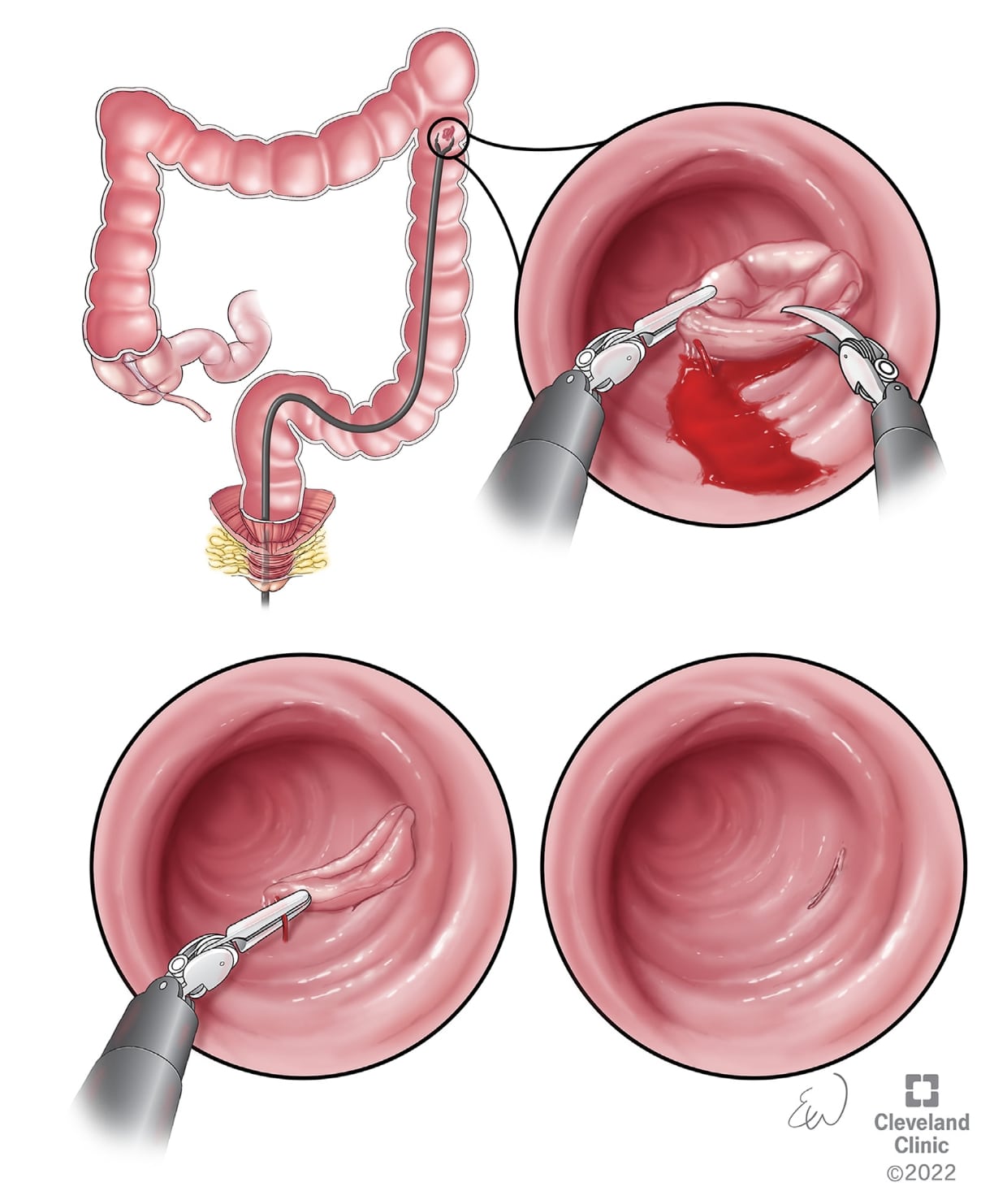

Supplement: Supplementary file 1 — Supplementary file1 (JPG 135 KB) [file 464_2026_12671_MOESM1_ESM.jpg]
